# Cost-Effectiveness of the Remote Primary Care and Psychiatry Model (MAP-PSI) for Depression in Youth Living in Rural and Indigenous Communities

**DOI:** 10.62641/aep.v54i4.2180

**Published:** 2026-08-15

**Authors:** Héctor Cabello-Rangel, Héctor Guillermo García-De Los Santos, Jorge Luis López-Jiménez, Marco Antonio Vázque-Rosales, Mauricio Israel Velázquez-Posada, Lina Díaz-Castro

**Affiliations:** ^1^Department of Research, Psychiatric Hospital Fray Bernardino Álvarez, 14080 Ciudad de México, Mexico; ^2^National Public Health Service, Ministry of Health, 78000 San Luis Potosí, SLP, Mexico; ^3^Department of Psychosocial Intervention Models, National Institute of Psychiatry Ramón de la Fuente Muñiz, 14370 Ciudad de México, Mexico; ^4^Faculty of Medicine, Cuauhtémoc University, 78000 San Luis Potosí, SLP, Mexico; ^5^General Directorate of Planning and Budget, Ministry of Health, 11800 Ciudad de México, Mexico; ^6^Department of Epidemiological and Psychosocial Research, National Institute of Psychiatry Ramón de la Fuente Muñiz, 14370 Ciudad de México, Mexico

**Keywords:** cost-effectiveness, depression, youth, mental health promotion

## Abstract

**Background:**

Community- and school-based interventions focused on mental health promotion and early detection of depressive symptoms have proven to be cost-effective. Our objective was estimated the cost-effectiveness ratio of the remote and pilot Primary Care and Psychiatry Model (MAP-PSI) compared with community usual care (CUC) in a rural municipality in Mexico.

**Methods:**

We analyzed all adolescents and young adults aged 15–25 years who received care between November 2023 and November 2024. Effectiveness measures included care-seeking behavior, school absenteeism prevented, suicide attempts avoided and suicides prevented. Direct and indirect costs including lost workdays, were considered. Incremental Costs-Effectiveness Ratios (ICERs) and deterministic sensitivity analyses were performed.

**Results:**

Of 1057 recruited participants, 932 completed screening (88.2%); mean age was 16.63 years ± standard deviation (SD) 1.53, and 60.51% were female. MAP-PSI delivered mental health care to 932 youths compared with 16 under CUC and was associated with the prevention of 12,195 school absenteeism days, at an incremental cost of $7.84 USD per absenteeism day avoided. Based on model projections, the intervention reduced expected suicide attempts from 13 to four cases; corresponding to nine suicide attempts avoided at an estimated incremental cost of $10,622.10 USD per suicide attempt avoided. Sensitivity analyses suggested that lower intervention effectiveness substantially increased ICERs under conservative scenarios.

**Conclusions:**

The MAP-PSI is a cost-effective strategy for reducing suicidal behavior and school absenteeism in young people with depressive symptoms living in rural and Indigenous communities.

## Introduction

Anxiety and depressive disorders constitute the greatest economic burden among the mental disorders analyzed in the Mexican population with and without social security. This burden is due both to their high prevalence and to the total volume of health resources required for their care. This trend is consistent with global evidence showing that depressive and anxiety disorders are leading contributors to disease burden worldwide [1], as well as with economic studies demonstrating their substantial cost implications in specific settings such as the United States [2].

Depression care involves intensive use of resources at all levels of the health system, including medical consultations, medications and, in many cases, hospitalization. While generic antidepressants are low-cost in most countries, the total cost of treatment varies widely depending on the type of drug, frequency of medical visits, and ancillary services required [3]. In addition, a significant proportion of the total cost corresponds to indirect costs, mainly due to loss of productivity, absenteeism from work, and disability, which substantially increases the economic burden of the disease [4].

In socially vulnerable populations, access to care and availability of social support are often limited [5]. Structural determinants such as geographic isolation, poverty, limited health infrastructure, and persistent cultural and linguistic barriers hinder timely access to care, as documented in global reports and in studies of indigenous and other marginalized populations [5, 6]. Additional social factors such as bullying have shown a significant association with increased depression, especially following the Coronavirus Disease 2019 (COVID-19) pandemic [7].

Globally, the burden of major depressive disorders increased markedly between 1990 and 2021, showing a 56.36% increase in prevalence, particularly among young people aged 20–24 years. The consequences of violence, bullying, and child sexual abuse contribute significantly to this burden [8]. Although the global suicide rate among adolescents and young adults has shown a declining trend during the same period, in Mexico, an increasing trend in suicide mortality was observed in recent decades [9].

Economic evaluations of preventive mental health interventions suggest that early community-based strategies may be cost-effective. For example, school based cognitive behavioral therapy programs have demonstrated favorable cost-benefit ratio and improvement in Quality-Adjusted Life Years (QALYs) [10]. In low-resource settings where young people face structural barriers to accessing formal care, schools have become key spaces for accessible and supportive mental health services. Moreover, parental involvement significantly improves outcomes [11].

Recent evidence suggests integrated remote intervention models, combining psychoeducation with psychological services and psychiatric care, may represent a scalable approach to improve mental health outcomes in underserved communities [12, 13]. When adapted to local cultural and linguistic contexts, these models can promote resilience, improve mental health literacy and reduce depressive symptoms [14, 15].

The Primary Care and Psychiatry Model (MAP-PSI) is a community-based intervention implemented in rural communities of San Luis Potosí, Mexico, designed to reduce depressive symptom burden and improve timely access to care among adolescents and young adults. The model integrates mental health promotion, psychological care, and telepsychiatry consultation within primary care settings. Conceptually, the intervention operates through a stepped-care pathway that enables early detection and management of depressive symptoms in community settings, potentially preventing clinical deterioration and reducing the need for costly psychiatric hospitalization. The expected impact of this intervention includes improved care-seeking behavior, prevention of suicide attempts, reduction in suicides, and decreased school absenteeism.

The objective of this study was to evaluate the cost-effectiveness of the MAP-PSI compared with community usual care (CUC) by applying an economic evaluation framework. Specifically, the study estimated the Incremental Cost-Effectiveness Ratio (ICER) associated with the MAP-PSI intervention.

We hypothesize that the integrated MAP-PSI approach will yield a lower ICER than CUC while generating positive outcomes at clinical, educational, social and economic domains; pointing to intercultural telepsychiatry as a policy-relevant strategy for reducing global inequality in youth mental health care, particularly in underserved rural contexts.

## Materials and Methods

### Study Design

A quasi-experimental implementation pilot study was conducted from November 2023 to November 2024 in the municipality of Ciudad Fernández (CDFDZ), San Luis Potosí, Mexico, complemented by a decision-analytic cost-effectiveness model, allowing comparison between the MAP-PSI intervention, CUC, and psychiatric hospital care for severe cases. The economic evaluation estimates the ICER associated with the intervention using observed service utilization and modeled counterfactual scenarios.

### Study Setting and Population

This study was conducted in the state of San Luis Potosí, one of the 32 federal entities of Mexico, selected due to its high proportion of Indigenous population (23%) and marked social vulnerability. San Luis Potosí has 2.8 million inhabitants, of whom 17% (around 480,000) are adolescents and young adults aged 15 to 24 years [16]. Fieldwork was carried out in the municipality of CDFDZ, located in the central region of the state. According to official demographic data, the population aged 15 to 24 years in CDFDZ totals 8341 individuals; however, only 39% of this age group remains enrolled in formal education [17].

At the time of the pilot study, CDFDZ lacked specialized mental health services, and local epidemiological surveillance systems. Consequently, national indicators were used to contextualize the mental health burden. According to the 2022 National Health and Nutrition Survey (ENSANUT) in México, 17% of the population reported depressive symptoms in the last month [18], and about 10% may develop a mental disorder that requires psychiatric care. The suicide rate in Mexico is estimated to be 6.8 per 100,000 inhabitants per year [16]. Based on the strong association between major depressive disorder and suicidal behavior, 10% was used as a conservative modeling assumption to represent the proportion of severe depression cases that may progress to suicide attempts without timely treatment [19]. These indicators suggest that between 2 and 5 suicides occur annually in CDFDZ, a non-trivial number that supports the need for accessible community-based responses.

### Sample

The study used a non-probabilistic, consecutive sampling strategy. Inclusion criteria were adolescents and young adults aged 15–25 years, of both sexes, residing in communities within the municipality of CDFDZ, and who provided informed consent (and parental consent when applicable) to participate in the study. Participants were excluded if they did not complete the study questionnaires required for the analysis. A total of 1505 individuals attended the mental health promotion sessions, 1098 completed the Knowledge, Attitudes, and Practices (KAP) questionnaire, and of these 1057 consented to the screening, and 932 answered the sociodemographic questionnaires and Patient Health Questionnaire-9 (PHQ-9). Caregiver data were obtained only for participants aged <18 years.

The CUC comparator was based on historical service utilization records from the Community Health House in CDFDZ, where 16 adolescents and young adults received mental health consultations between 2020 and 2022.

### Effectiveness Outcomes

Effectiveness was measured in terms of four indicators:

(1) Improvement in mental health care-seeking, evaluated through changes in knowledge and attitudes measured with the KAP questionnaire. This questionnaire was derived from the original instrument described in [20] and culturally adapted for application in the target study population. Depressive symptoms were assessed using the PHQ-9, which consists of nine items scored from 0 to 3, and a total score ranging from 0 to 27. Depression severity was classified according to standard cut-off points: minimal (0–4), mild (5–9), moderate (10–14), moderate-severe (15–19), and severe (20–27). For the cost-effectiveness analysis, PHQ-9 classifications were based on initial screening data to reflect real-world implementation conditions. More restrictive analytical datasets requiring complete item responses were used for separate validation analyses and are reported elsewhere [21].

(2) Suicide attempts prevented, estimated through the decision-analytic model based on severe depressive symptom cases and suicide-risk assumptions.

(3) Suicides prevented, estimated through the same model.

(4) School absence days prevented, estimated from self-reported absenteeism among participants with moderate-to-severe depressive symptoms over the analytic period.

### Interventions and Comparator

For the present study, the following alternatives were considered.

(1) The pilot and remote MAP-PSI:

The MAP-PSI is an operational mental health intervention implemented to reduce depressive symptom burden and improve timely access to care among adolescents and young adults aged 15–25 years.

MAP-PSI combines community mental health promotion with a stepped clinical pathway. It integrates three core clinical components: (1) psychoeducation, (2) psychological care, and (3) specialized psychiatric care delivered through telepsychiatry, supported by local service strengthening (training, referral pathways, and infrastructure).

Participants considered in this study first received psychoeducation (n = 1057) and then they consented to be screened using the PHQ-9 scale (n = 932). Individuals with mild to moderate depressive symptoms were offered structured psychological sessions, while severe cases were referred for remote psychiatric consultation. Psychoeducation sessions focused on mental health literacy, recognition of depressive symptoms, coping strategies, and family support. Sessions were delivered by trained health personnel and community promoters and complemented with remote psychiatric evaluation when clinically required. The approach is culturally responsive through the inclusion of bilingual Xi’iui–Spanish community promoters and locally adapted educational materials.

The pilot phase was organized into three sequential implementation stages:

Stage 1—Exploration (March–September 2023): Mapping of community needs, stakeholders’ identification, universal and targeted promotion sessions for youth, community and caregiver sensitization, and PHQ-9 screening.

Stage 2—Installation (April–October 2023): MAP-PSI established the operational base by adapting an existing primary care facility (Community Health House) into the MAP-PSI Community Mental Health House, equipped for telemedicine and training of personnel.

Stage 3—Initial implementation (November 2023–November 2024): Delivery of the service package including psychoeducation, psychological interventions, and telepsychiatry consultations during the pilot implementation period reported in this study.

**Fig. 1.**
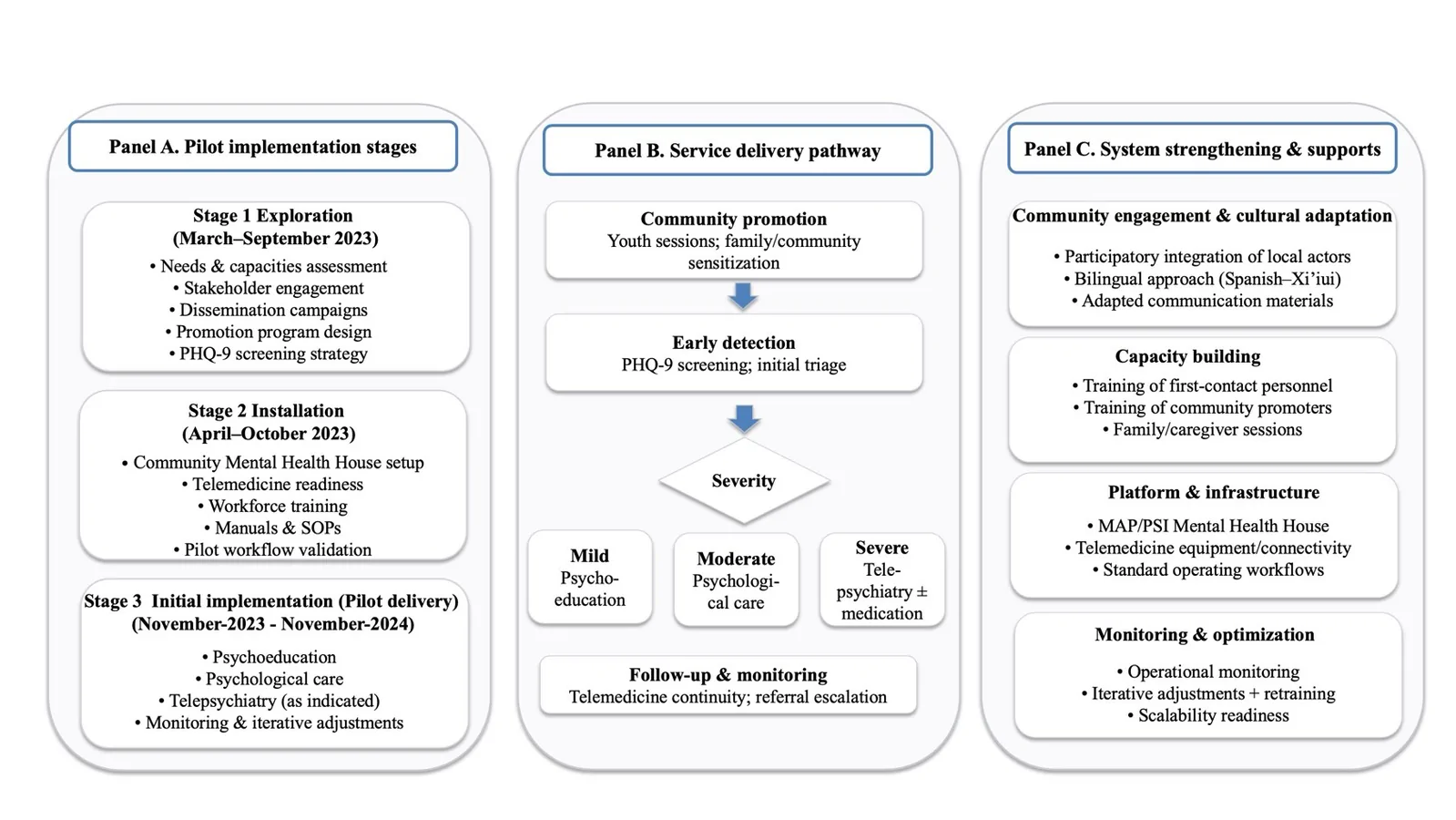
**MAP-PSI pilot implementation framework and service delivery pathway**. MAP-PSI, Primary Care and Psychiatry Model; PHQ-9, Patient Health Questionnaire-9.

A schematic overview of the MAP-PSI, its implementation stages, and its service delivery components is presented in Fig. 1.

(2) Community usual care:

CUC represents the standard pathway available for mental health problems in rural communities of San Luis Potosí. Care is typically provided by a general practitioner and referral to the psychiatric hospital located in the state capital when specialized care is required. From August 2020 to November 2022, the Community Health House in CDFDZ provided 5354 general medical consultations, of which 163 (3.04%) corresponded to a mental health condition. Anxiety and depression constituted 73% of all cases, the rest were due to schizophrenia, bipolar disorder and autism. Only 16 people were between the ages of 12 and 25 (9 women and 7 men). Data on service utilization were obtained from unpublished administrative records of the Community Health House in CDFDZ covering the period 2020–2022.

(3) Psychiatric hospital care:

For severe cases requiring specialized treatment, care is provided at the psychiatric hospital located in the state capital. This level of care may include psychiatric evaluation, pharmacological treatment, and hospitalization in cases involving suicide attempts or severe depressive episodes. Hospital treatment costs were included in the economic model as the reference cost for severe cases requiring inpatient care, applicable to both intervention and usual care scenarios.

### Statistical Analysis

Costs were estimated from a health system perspective, incorporating both direct and indirect costs.

Direct costs included expenditures related to mental health promotion activities, psychoeducation, psychology and medical care at general and specialized levels. Unit costs were estimated based on minimum daily wages according to the Ministry of Health wage scale [22]. A market survey carried out in CDFDZ, San Luis Potosí, Mexico, in 2024, by the research team, estimated the average cost of commonly used antidepressants (fluoxetine or sertraline) at $0.30 USD per tablet (See Table 1).

**Table 1.** **Unit costs of health care activities for a case of depression**.

Activity	Personnel	Daily wage (USD)	Unit cost per activity (USD)	Source
Promotion and screening	Nurse	$47.79	$3.98	Ministry of Health Wage Scale, 2024
Psychoeducation	Promoter	$37.31	$1.87	-
Psychotherapy	Psychologist	$60.43	$7.55	-
General medical consultation	General practitioner	$76.44	$6.37	-
Lost productivity (per day)	General salary	$14.26	$14.26	-
Specialized psychiatric care	Psychiatrist	$90.64	$11.33	-
Pharmacotherapy (antidepressant)	Box	$8.58	$0.30	Market survey

Unit costs were estimated using the Ministry of Health Wage Scale (2024) and time per activity. Pharmacotherapy cost represents the average price per antidepressant tablet (fluoxetine/sertraline) based on a local market survey. Productivity losses were valued using the daily minimum wage. All costs are expressed in USD (exchange rate: $17.46 MXN = $1 USD; January 2024).

Intervention costs were modeled as a function of the number of sessions delivered per participant. The cost of one day of psychiatric hospitalization was estimated at $90.64 USD, adjusted for inflation (85.95%) as of May 2025 [23, 24]. Indirect costs, including transportation and lost workdays, were estimated using the 2024 minimum wage for San Luis Potosí ($14.26 USD per day) according to the National Commission on Minimum Wages, Ministry of the Interior [22].

Total cost per patient was calculated by multiplying unit cost (Table 1) by the frequency of service utilization under each care modality, including CUC, MAP-PSI, and psychiatric hospital care. This estimate incorporated both direct healthcare costs (consultations, medications, and hospitalization) and indirect costs (transportation and productivity losses), allowing comparison of the additional cost associated with the intervention relative to the usual care pathway.

The ICER was calculated as the ratio of the difference in costs to the difference in effectiveness between MAP-PSI and CUC.

For the sensitivity analysis, the values of the variables selected were the base case and the most optimistic scenario. Subsequently, the analysis was reformulated using a range of possible values to generate the best (worst) possible scenario [25]. This analysis excludes the costs of telepsychiatry equipment. Costs expressed in American dollar, using an exchange rate of $17.46 MXN per $1 USD (January 2024).

## Results

The results are presented in two parts: observed outcomes from the MAP-PSI implementation and modeled projections derived from the economic evaluation.

### Observed Outcomes During MAP-PSI Implementation

Of the 1057 participants recruited, 932 participated in the screening process, the majority were female (60.51%), with a mean age of 16.63 years (standard deviation [SD] = 1.53, observed range: 15–25 years). Seven participants reported speaking an Indigenous language. As for caregivers, 806 participants had a mean age of 43.28 years ± 7.88, 65.5% were women. Mean household income was $628.5 USD, and the mean number of dependents per household was 3.75 (SD = 1.52). Participants sociodemographic characteristics are summarized in Table 2.

**Table 2.** **Sociodemographic characteristics of participants under MAP-PSI care**.

Characteristic			n (%) or mean ± SD	
Age [(years), mean ± SD]			16.63 ± 1.53	
Gender [(n = 932), n (%)]				
	Male		368	39.49
	Female		564	60.51
Indigenous language speakers [(n = 932), n (%)]			7	0.75
Caregivers (n = 806), n (%)				
	Father		263	32.63
	Mother		528	65.5
	Both parents		15	1.86
Household income, mean ± SD			628.5 ± 681.2^*^	
Number of dependents, mean ± SD			3.75 ± 1.52	
Current educational enrollment [(n = 806), n (%)]				
	High school-first year		319	39.58
	High school-second year		238	29.53
	High school-third year		180	22.33
	University		69	8.56
Occupational activity [(n = 923), n (%)]				
	Students only		787	85.27
	Students and formal employee		44	4.76
	Students and freelance workers		59	6.39
	Students and household activities		12	1.30
	Students and the unemployed due to health reasons		2	0.22
	Students and unemployed for other reasons		19	2.06
PHQ-9 categories [(n = 817), n (%)]				
	Minimal (0–4)		313	38.31
	Mild (5–9)		233	28.52
	Moderate (10–14)		145	17.75
	Moderate-severe (15–19)		67	8.20
	Severe (20–27)		59	7.22
Type of care received, n (%)				
	Promotion and screening		932	100
	Clinical subsample attending Community Health House (n = 387)			
		Psychoeducation sessions	32	8.3^**^
		Clinical psychology	160	41.3^**^
		Telepsychiatry consultation	195	50.4^**^

SD, standard deviation; PHQ-9, Patient Health Questionnaire-9. Data are presented as n (%) or mean ± SD. Indigenous language speakers, refers to participants who self-reported speaking an Indigenous language. ^*^Household income expressed in American dollar, using an exchange rate of $17.46 MXN per $1 USD (January 2024). ^**^Percentages for type of care received refer to participants undergoing second PHQ-9 screening and clinical allocation.

During the implementation period, 932 individuals accessed mental health care under the MAP-PSI, compared with only 16 adolescents under CUC.

### Modeled Outcomes From the Economic Evaluation

Economic projections were derived from the decision-analytic model comparing MAP-PSI with CUC and psychiatric hospital care.

The average cost per treated case under the MAP-PSI was $102.57 USD higher than under CUC because the intervention includes a broader set of services, including screening, psychoeducation, psychological care, and telepsychiatry consultation. However, this cost remained lower than hospital-based treatment at the psychiatric facility (Table 3).

**Table 3.** **Comparison of cost of CUC versus MAP-PSI care**.

		Cost per unit of care	Number of contacts/sessions/days			Cost of care per patient		
			Usual care	MAP-PSI	HPSnLP	Usual care	MAP-PSI	HPSnLP
Case of depression								
	Promotion (general nurse)	$3.98	1	2	3	$3.98	$7.96	$11.95
	Psychoeducation (promoter)	$1.87	0	5	3	$0.00	$9.33	$5.60
	Psychotherapy (clinical psychologist)	$7.55	0	8	1	$0.00	$60.43	$7.55
	Pharmacotherapy	$0.30	180	180	180	$55.15	$55.15	$55.15
	General practitioner	$6.37	1	10	0	$6.37	$63.70	$0.00
	Psychiatrist	$11.32	0	0	2	$0.00	$0.00	$22.66
	Transport	$14.26	1	0	14.5	$14.25	$0.00	$206.21
	Days lost from work (family)	$14.26	1	0	6	$14.25	$0.00	$85.47
	Total case					$94.00	$196.57	$394.59
	Total population (n = 932)					$87,606.40	$183,205.26	$367,758.34
Suicide attempt (SA)								
	Pharmacotherapy	$0.30	180	180	180	$55.15	$55.15	$55.15
	Hospital (day)	$92.44	5	5	5	$462.21	$462.21	$462.21
	Total case plus SA					$611.36	$713.94	$911.94
	Total population: CUC, n = 13; MAP-PSI, n = 4; HPSnLP, n = 2.					$7947.73	$2855.75	$1823.87

Costs are presented in USD. Unit costs correspond to the cost per activity/session/day (in Table 1). Number of contacts/sessions/days reflects the assumed service utilization under each care pathway. Cost of care per patient was estimated as unit cost × frequency of service use. CUC, community usual care; MAP-PSI, Primary Care and Psychiatry Model; HPSnLP, psychiatric hospital care in San Luis Potosí. Components may not sum to totals due to rounding.

The incremental cost of associated with increased mental health services utilization was estimated at $118 USD per additional participant receiving mental health care. If equivalent care was delivered through the psychiatric hospital, total cost would increase substantially due to transportation and hospitalization expenses (Table 4).

**Table 4.** **Cost-effectiveness analysis of MAP-PSI versus CUC and hospital care**.

Intervention	Cost of the intervention (USD)	Participants receiving mental health care (n)	Average cost per participant receiving care (USD)	School absenteeism (day)	Average cost per day of absenteeism (USD)	Suicides averted (n)	Average cost per suicide averted (USD)
CUC (P1)	$87,606.40	16	$5,475.40	14905	$5.88	0	
MAP-PSI (P2)	$183,205.26	932	$197.57	2710	$67.60	4	$45,801.31
HPSnLP (P3)	$367,758.34	932	$394.60	2710	$135.70	1	$367,758.34
Incremental cost-effectiveness analysis using CUC (P1) as comparator							
Comparison	ΔCost (USD)	Additional participants receiving mental health care (n)	ICER (USD per additional participant receiving care)	School absenteeism avoided (days)	ICER (USD per day avoided)	Additional suicides averted (n)	ICER (USD per suicide averted)
Δ P2 P1	$95,598.86	916	$104	12195	$7.84	4	$23,899.71
Δ P3 P1	$280,151.94	916	$306	12195	$22.97	1	$280,151.94

Costs are expressed in USD (exchange rate: $17.46 MXN = $1 USD; January 2024). P1 = community usual care (CUC); P2 = Primary Care and Psychiatry Model (MAP-PSI); P3 = psychiatric hospital care in San Luis Potosí (HPSnLP). Outcomes included participants receiving care, school absenteeism (days), and suicides averted (model-based estimates). Incremental Cost-Effectiveness Ratios (ICERs) were calculated as incremental divided by incremental effect (ΔCost/ΔEffect) using CUC (P1) as the comparator.

In the economic model, school absenteeism under CUC was estimated at 14,905 days, compared with 2710 days under MAP-PSI. Thus, MAP-PSI was associated with 12,195 school absenteeism days avoided. The average cost per absenteeism day was $5.88 USD under CUC and $67.60 USD under MAP-PSI care, however, when expressed incrementally, the cost was $7.84 USD per absenteeism day avoided. When efficiency is expressed in terms of cost per day of absenteeism prevented, the adjusted cost under the MAP-PSI care was $7.84 USD per day, a value that quadruples if the patient attends the psychiatric hospital (Table 4).

### Suicide Risk Prevented

Suicide risk projections were estimated based on PHQ-9 severity categories, 271 participants had moderate to severe depressive symptoms, including 126 participants with moderate-severe to severe depressive symptoms associated with an increased risk of suicide. Based on the decision-analytic model, approximately 10% of severe depression cases may attempt suicide in the absence of timely care [19]. An estimated 13 suicide attempts could occur under CUC, whereas MAP-PSI reduced the expected number to four cases, corresponding to nine suicide attempts avoided at an estimated incremental cost of $10,622.10 USD per suicide attempt avoided (Table 5).

**Table 5.** **Cost-effectiveness analysis (ICER) of suicide attempt averted**.

	Cost (USD)	Suicide attempt (n)	Average cost per suicide attempt (USD)
CUC (P1)	$87,606.40	13	$6,738.95
MAP-PSI (P2)	$183,205.26	4	$45,801.31
HPSnLP (P3)	$367,758.34	2	$183,879.17
Incremental cost-effectiveness analysis			
Comparison	ΔCost (USD)	Suicide attempts avoided (n)	ICER (USD per suicide attempt avoided)
ΔP2 vs P1	$95,598.86	9	$10,622.10
ΔP3 vs P1	$280,151.94	11	$25,468.36

Costs are expressed in USD (exchange rate: $17.46 MXN = $1 USD; January 2024). P1 = community usual care (CUC); P2 = Primary Care and Psychiatry Model (MAP-PSI); P3 = psychiatric hospital care in San Luis Potosí (HPSnLP). Suicide attempts are model-based estimates derived from severe depression cases. Incremental Cost-Effectiveness Ratios (ICERs) were calculated as ΔCost/ΔEffect using P1 as the comparator and reported per suicide attempt avoided.

### Sensitivity Analysis

Sensitivity analysis showed that maintaining the MAP-PSI intervention remained economically favorable compared with CUC. Although the estimated cost of preventing suicide attempt and school absenteeism increased under this conservative scenario, the cost remains reasonable up to 50% effectiveness. (See Table 6).

**Table 6.** **Sensitivity analysis of MAP-PSI cost-effectiveness**.

Intervention	Cost (USD)	Participants receiving mental health care (n)	Additional participants receiving care (n)	ICER (USD per additional participant)	School absenteeism avoided (days)	ICER (USD per day avoided)	Suicide attempts avoided (n)	ICER (USD per suicide attempt avoided)
MAP-PSI (90%)	$95,598.86	839	823	$116.16	10,975	$8.71	8	$11,949.85
MAP-PSI (50%)	$95,598.86	466	450	$212.44	6097	$15.68	5	$19,119.77
MAP-PSI (25%)	$95,598.86	233	217	$440.55	3049	$31.35	2	$47,799.42

Costs are expressed in USD (exchange rate: MXN $17.46 = USD $1.00; January 2024). MAP-PSI, Primary Care and Psychiatry Model. Sensitivity scenarios were estimated by proportionally reducing the incremental effectiveness of MAP-PSI compared with cost of CUC. Incremental Cost-Effectiveness Ratios (ICERs) were calculated as ΔCost/ΔEffect using cost of CUC as comparator.

## Discussion

This study suggests that the remote MAP-PSI care may be more cost-effective than CUC for reducing the burden of depression and its social consequences in rural and Indigenous communities characterized by persistent structural barriers to mental health care access [2, 5]. This effectiveness is due not only to the availability of remote interventions, but also to their cultural integration through bilingual materials in Spanish and Xi’iui and the involvement of community promoters trained in mental health.

From a social impact perspective, the implementation of the model strengthened local capacities through the involvement of first-contact health professionals, the family members, caregivers, and community promoters. This participatory and contextualized strategy facilitated the appropriation of the model by the community, a fundamental factor for sustainability in resource-limited settings [11].

The MAP-PSI also generated substantial educational benefits, such as reducing school absenteeism by 12,195 days, which is directly associated with the timely treatment of depressive symptoms. Since lower levels of education are a recognized risk factor for suicide attempts, reductions in school absenteeism carry long-term benefits, ensuring learning continuity represents a viable strategy for suicide prevention [26].

These findings are consistent with previous economic evaluations showing that prevention and promotion interventions in school and community settings can be cost-effective approaches to mitigate youth depression [10].

In terms of public health, suicide prevention and the reduction of suicide attempts in young people are key findings that reinforce the importance of early, community-based, and evidence-based interventions to reduce preventable suicides from psychiatric causes [8]. This is particularly important in vulnerable groups such as adolescents in rural communities where the prevalence of desire to die and suicidal thoughts has been reported to be high, for example, in Peru, a prevalence of 21.4% was reported [27]. Also, these individuals’ social difficulties are often more extreme, including poverty, poor health and well-being, limited health literacy, substance abuse, or abuse by parents or guardians [28].

This pilot implementation in CDFDZ offers valuable guidance on how to structure and implement cost-effective mental health care models in low- and middle-income countries. The results of this intervention can support the outcomes of similar projects, such as the Electronic Surveillance System for the Early Notification of Community-based Epidemics (ESSENCE) program in India, which aims to develop and evaluate a digital program to train non-specialist health workers to provide treatment for depression [29].

With technical support, measurable results, and local acceptance, the MAP-PSI is likely to be scaled up to other regions with similar characteristics, as even with a 50% performance rate, it remains cost-effective. In this regard, the need to implement sustainable, scalable, and cost-effective programs in the immediate future has been highlighted, given the scarcity of community-based mental health interventions [30]. Mental health promotion and preventive intervention have demonstrated favorable cost-effectiveness profiles in reducing depressive symptoms and suicidal behaviors in adolescents and adults [31]. Likewise, the inclusion of digital tools in the promotion and detection of depressive symptoms is cost-effective [32].

This evaluation has limitations. First, telepsychiatry equipment costs were not included because such equipment is not standard in Mexican primary care units; dedicated feasibility analyses are required to support comprehensive inclusion. Second, PHQ-9 score changes were not reported, limiting interpretation of symptom-level improvement. Another limitation of this study relates to the quasi-experimental nature of the pilot implementation. Participation in the MAP-PSI intervention occurred through voluntary engagement following community outreach and screening activities. Therefore, the possibility of selection bias and self-selection into care cannot be excluded, as individuals seeking care may differ systematically from those who do not seek services. Additionally, contextual factors such as socioeconomic status, family support, and educational environment may act as potential confounders influencing mental health outcomes. On the other hand, in relation to the uncertainty associated with several parameters used in the economic model, the proportion of severe depression cases assumed to progress to suicide attempts, and the extrapolation of school absenteeism reductions were derived from modeling assumptions informed by epidemiological evidence and pilot data. These parameters may vary across populations and contexts, and therefore the estimates presented in this study should be interpreted as projections within the decision-analytic framework rather than precise predictions of real-world outcomes. Moreover, the number of Indigenous language speakers in the sample was small (n = 7); therefore, conclusions regarding Indigenous communities should be interpreted cautiously and require further evaluation in larger Indigenous populations. Future evaluations should incorporate longitudinal clinical outcomes and quality-of-life metrics to strengthen comparability with other cost-effectiveness of studies.

## Conclusions

The remote MAP-PSI based on mental health promotion and early detection of depressive symptoms in adolescents living in rural areas is cost-effective in terms of reducing suicidal behavior and school absenteeism, both results with long-term effects. By addressing school failure, and improving access to timely care, the model may also reduce longer-term risks of productivity loss and sustained mental health burden.

## Availability of Data and Materials

The datasets used and/or analyzed during the current study are available from the corresponding author on reasonable request.
